# Text mining for identifying topics in the literatures about adolescent substance use and depression

**DOI:** 10.1186/s12889-016-2932-1

**Published:** 2016-03-19

**Authors:** Shi-Heng Wang, Yijun Ding, Weizhong Zhao, Yung-Hsiang Huang, Roger Perkins, Wen Zou, James J. Chen

**Affiliations:** Division of Bioinformatics and Biostatistics, National Center for Toxicological Research, U.S. Food and Drug Administration, 3900 NCTR Road, HFT-20, Jefferson, AR 72079 USA; Graduate Institute of Biostatistics, China Medical University, No. 91, Xueshi Rd, Taichung City, 40402 Taiwan; National Applied Research Laboratories, National Center for High-Performance Computing, No. 7, R&D 6th Rd., Hsinchu Science Park, Hsinchu City, 30076 Taiwan

**Keywords:** Topic model, Text mining, Adolescent, Substance use, Depression

## Abstract

**Background:**

Both adolescent substance use and adolescent depression are major public health problems, and have the tendency to co-occur. Thousands of articles on adolescent substance use or depression have been published. It is labor intensive and time consuming to extract huge amounts of information from the cumulated collections. Topic modeling offers a computational tool to find relevant topics by capturing meaningful structure among collections of documents.

**Methods:**

In this study, a total of 17,723 abstracts from PubMed published from 2000 to 2014 on adolescent substance use and depression were downloaded as objects, and Latent Dirichlet allocation (LDA) was applied to perform text mining on the dataset. Word clouds were used to visually display the content of topics and demonstrate the distribution of vocabularies over each topic.

**Results:**

The LDA topics recaptured the search keywords in PubMed, and further discovered relevant issues, such as intervention program, association links between adolescent substance use and adolescent depression, such as sexual experience and violence, and risk factors of adolescent substance use, such as family factors and peer networks. Using trend analysis to explore the dynamics of proportion of topics, we found that brain research was assessed as a hot issue by the coefficient of the trend test.

**Conclusions:**

Topic modeling has the ability to segregate a large collection of articles into distinct themes, and it could be used as a tool to understand the literature, not only by recapturing known facts but also by discovering other relevant topics.

**Electronic supplementary material:**

The online version of this article (doi:10.1186/s12889-016-2932-1) contains supplementary material, which is available to authorized users.

## Background

Adolescent substance use is a major public health problem. Alcohol, tobacco, and drug use in adolescence are risk factors that portend lifelong negative consequences, such as substance use disorder in adulthood, greater welfare dependence, unemployment and lower life satisfaction [1, 2]. Considerable research has been devoted to understanding risk and protective factors related to adolescent substance use. Interactions within the family and peer relationships are critical social contexts to precipitate adolescent substance use [3]. Adolescent depression is also a major social problem. It has been shown to increase suicide attempts [4], increase the likelihood of substance use for self-medication [5], and negatively affect mental and physical health well into adulthood. The key risk factors of depression in adolescents include family history, psychosocial stress, genetic and environmental interaction, and parental factors and peer influence [6].

There are many adolescents suffering from substance use and depression, which tend to co-occur [7, 8]. An excellent review has discussed the co-occurring mental and substance use disorders and the neurobiological interface between them [9]. Genetic and environmental vulnerability factors, such as family and social influences, are associated with both substance use and psychiatric disorders. Chronic stress plays an important role in the bridging constructs between psychiatric and substance use disorders. There is a reciprocal relation between substance use and depression, and several studies have explored the mechanisms behind the association between them [10–14]. Better understanding of the initiation of adolescent substance use and the connection between substance use and depression may help inform prevention efforts.

To date, a large number of articles that focus on adolescent substance use or adolescent depression have been published. It is time-consuming and labor-intensive to extract and understand the information of these cumulated collections. Although the review articles perform a literature search and then summarize the findings, they usually focus on a specific issue. Text mining, a process of deriving patterns and trends from text, can be used as an alternative approach to gain a broad understanding of an entire dataset and to explore the dynamics of the study issues [15]. Text mining has been proposed as a screening process for identifying relevant studies expeditiously in systematic reviews [16].

Text mining applied techniques from machine learning and computational statistics to find important patterns in text data. There are many different approaches to analyze text data; previous studies have provided overviews of these text mining methods [17, 18]. Many applications of text mining in literature databases, such as PubMed and Medline, have been reviewed, and the benefits and challenges have been discussed [19–22]. The scientific literature could be a potential source of new knowledge [23]. Swanson [24, 25] has shown that manually connecting concepts between journal articles could extract some hidden relationships, which were confirmed by preceding experimentation. Two approaches, natural-language processing-based and statistical co-occurrence-based, have been used to extract entity relationships form scientific literatures. The natural-language processing-based approach usually extracts relationships within a document, while the statistical co-occurrence-based approach identifies co-occurrence structures in a set of documents.

Topic modeling is a widely used probabilistic modeling for text mining offering a computational tool to uncover topics capturing meaningful structure among collections of documents [26]. The objectives of topic modeling are to identify the topics referred to in a document, as well as to uncover the latent themes in collections of documents. This algorithm has been applied to help organize and understand scientific articles [26, 27], drug safety databases [28, 29], and social media [30]. In this study, we applied topic modeling to perform text mining on the published articles about adolescent substance use and depression to discover hidden textual patterns. We then performed trend analysis to explore the dynamics of proportion of topics and hierarchical clustering analysis to cluster similar topics.

## Methods

### Data set

We searched and retrieved the articles about adolescent substance use or adolescent depression in PubMed. Medical subject headings (MeSH) is a controlled vocabulary of pre-defined terms and is annually updated. MeSH terms are utilized for the purpose of indexing journal articles and can be served as a thesaurus facilitating searching in PubMed database. In some cases, MeSH terms may not fully represent the interested themes during the study, we used keywords to search for relevant articles. For adolescent substance use, we used four sets of keywords to search titles or abstracts: 1. adolescent(s) and substance; 2. adolescent(s) and alcohol; 3. adolescent(s) and tobacco; and 4. adolescent(s) and marijuana. The search criterion for adolescent depression was keywords adolescent(s) and depression in titles or abstracts. A total of 17,723 abstracts published from 2000 to 2014 were retreived and downloaded. The numbers of abstracts from adolescent substance use and adolescent depression were 11,563 and 7,268, respectively, with 1,108 overlapping abstracts.

To preprocess the text data, the general words, such as background, aim, method, result, conclusion, stop words, and numerical digits, were eliminated. In addition, adolescent and adolescents were also removed from the dataset to avoid poor discriminative information due to their presence in almost all abstracts retrieved. The preprocessing was performed by using MALLET (MAchine Learning for LanguagE Toolkit) [31], which is an open-source Java-based package for statistical natural language processing, topic modeling, and other machine learning applications to text.

### Topic modeling

Latent Dirichlet allocation (LDA) [32], one of the most popular topic modeling algorithms, was performed to pursue text mining of the corpus of abstracts. LDA is a hierarchical Bayesian approach; it learns a set of thematic topics from words that tend to occur together in documents. A single topic can be described as a multinomial distribution of words, and a single document can be described as a multinomial distribution of latent topics. The model uses the observed documents and words to infer the hidden topic structure, creating per-document topic distributions, *P(topic|document)*, and per-topic word distributions, *P(word|topic)*.

We used LDA in Mallet [31] to carry out Gibbs sampling [27] to obtain the posterior samples which were used to infer hidden topic structure. To obtain the sparse topic and word distributions for more interpretable topics, we choose small values on the Dirichlet hyperparameters, α (parameter of Dirichlet prior on the per-document topic distributions) and β (parameter of Dirichlet prior on the per-topic word distributions) equal to 0.1 and 0.01, respectively. It is a challenge to select an optimal number of topics. Though the perplexity-based method has been proposed, it may not result in clear interpretations. We, therefore, ran LDA with 5, 20, and 50 topics, and compared similarity and difference of content of topics obtained using the different models.

### Visualization of topics

For visualization of the content of topics, the most probable words to convey a topic meaning were listed with the RGB color model, an additive color model in which red (R), green (G), and blue (B) light are added together in various parameters to reproduce a broad spectrum of colors. The parameters of R, G, and B are all inversely proportional to the normalized probability of words, and the color is shaded in grey scale from black to white. The higher color depth indicates the higher probability. The RGB color model was plotted with Java language. The word clouds (https://www.jasondavies.com/wordcloud/) were also plotted to demonstrate the distribution of vocabularies over each topic. To make the visualization clear, we combined the singular and the plural into one word if they were both in the top 20 probable words for a given topic. The individual topics were presented as an unstructured set of word clouds, and the word size is proportional to the probability of the word within a topic, *P(word|topic)*.

### Dynamics and hierarchical clustering of topics

Each document was assigned to a topic with the highest probability, *P(topic|document)*; the frequency distributions of assigned topics were plotted for two sets of search results, from adolescent substance use and from adolescent depression. In addition, we analyzed the dynamics of these topics. A trend analysis on the proportion of each topic from 2000 to 2014 was conducted. A *p*-value smaller than Bonferroni-corrected alpha, 0.05/20 = 0.0025, was considered as statistically significant.

To explore the relationship between topics, we performed hierarchical clustering analysis to cluster topics based on the topic-word matrix, which was transformed to binary data with a 1/0 to indicate presence of a word in a given topic. The distance among topics was calculated based on the Jaccard distance and the average linkage method was applied with an agglomerative clustering algorithm to generate the cluster dendrogram. The hierarchical clustering analysis was conducted with R package. Topics containing similar words were assigned to the same clusters.

## Results

The 20 most probable words in grey scale of the topics in LDA with 5, 20, and 50 topics are listed in Additional files 1, 2, and 3, respectively. The topics extracted from LDA with k topics are named T0-T(k-1), e.g., T0, T1, T2, T3, and T4 for LDA with 5 topics. The cumulative probability of the 10 most probable words for LDA with 5 topics was smaller than that for LDA with 20 or 50 topics. A few words cannot convey a topic meaning sufficiently if a small number of topics is assumed. LDA with 5 topics identified themes referring to search keywords, such as substance, alcohol, tobacco, and depression. However, different issues were lumped together in the same topic, such as alcohol and brain research together in T0, alcohol and sexual experience in T3, and tobacco and alcohol in T4. Beyond recapturing themes referring to search keywords, LDA with 20 topics discovered some relevant issues, such as intervention and treatment program (T4, T12, and T15), family influence and peer network (T8 and T19), diet, physical activity, and obesity (T13), suicide (T16), and brain research (T18). LDA with 50 topics discovered additional themes, such as T3 and T23 referring to genetic and environmental influence, but also identified some topics referring to the same issue, such as T6 and T7 topics referring to depression, and T37 and T44 referring to alcohol drinking. We selected LDA with 20 topics with the following interpretation of results.

**Fig. 1:**
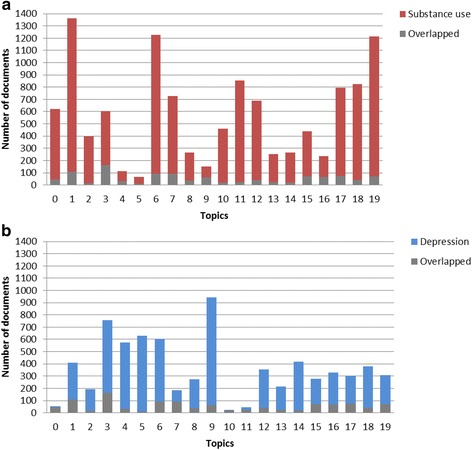
Distributions of assigned topics with the highest probability for documents. **a** abstracts searched by adolescent substance use; **b** abstracts searched by adolescent depression

**Fig. 2:**
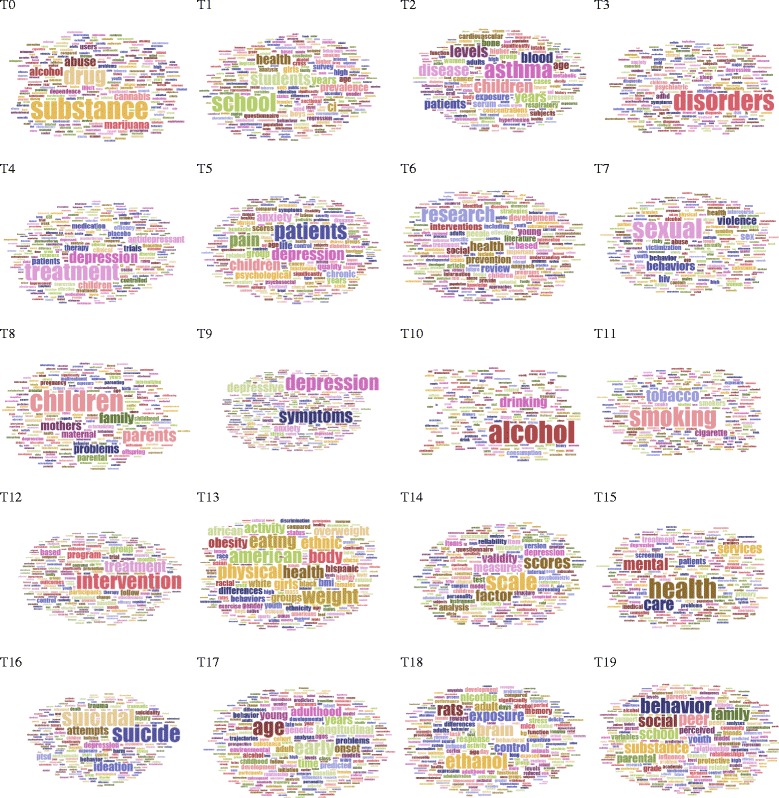
Word clouds for 20 topics

Distributions of assigned topics for the documents are shown in Fig. 1. The most ten probable words for each topic are listed in Table 1, and the corresponding word cloud for each topic is shown in Fig. 2. The most popular five topics among abstracts about adolescent substance use were T1, T6, T11, T18, and T19, which pertain to substance use, general research terms, tobacco smoking, brain research, and family factors and peer network, respectively. The results indicated the effort of many studies to explore the risk factors of adolescent substance use and the application of a cognitive model to study the harm of substance use.

**Table 1:** The most ten probable words in the topics of LDA with 20 topics

T0: substance use	substance drug abuse alcohol marijuana cannabis users dependence illicit treatment
T1: study	school students health ci prevalence girls boys age years high
T2: diseases	asthma children disease levels blood years patients age serum exposure
T3: psychiatric disorder	disorders psychiatric adhd sleep depression anxiety children symptoms clinical mdd
T4: treatment of depression	treatment depression children trials patients therapy medication placebo clinical controlled
T5: depression	patients pain depression children anxiety life group psychological chronic years
T6: general terms	research health review prevention interventions social young based development people
T7: sex and violence	sexual behaviors violence sex hiv health abuse victimization youth substance
T8: family	children problems parents family mothers maternal parental parent offspring childhood
T9: depression	depression symptoms depressive anxiety stress girls levels depressed coping social
T10: alcohol drinking	alcohol drinking consumption related binge heavy age drinkers drink frequency
T11: tobacco smoking	smoking tobacco smokers cigarette exposure smoke cigarettes current cessation nicotine
T12: intervention program	intervention treatment program group based follow participants control months outcomes
T13: eating, physical activity, obesity	weight american physical eating body ethnic health activity obesity girls
T14: questionnaire of depression	scale factor scores validity depression analysis items test reliability version
T15: health care services	health care mental services treatment patients screening primary medical problems
T16: suicide	suicide suicidal ideation ptsd attempts depression trauma injury behavior harm
T17: development	age early adulthood years longitudinal young time onset genetic adult
T18: brain research	ethanol brain rats exposure adult nicotine response memory stress mice
T19: family and peer	peer social family substance school parental behavior youth perceived protective

*adhd* attention deficit hyperactivity disorder, *mdd* major depressive disorder, *hiv* human immunodeficiency virus, *ptsd* post-traumatic stress disorder

The 5 most popular topics among abstracts about adolescent depression were T5 and T9 pertaining to depression, T3 for psychiatric disorders, T4 for treatment of depression, and T6 for general research terms. The results indicated that many studies focus on the efficacy of medication for depression, and explore the comorbidity between depression and other psychiatric disorders.

Among overlapping abstracts, the most 5 popular topics were T1, T3, T6, T7, and T17, which refer to substance use, psychiatric disorders, general research terms, sex and violence, and development from childhood to adulthood, respectively. The results indicated that many studies explored the reciprocal relation between substance use and depression, and studied the role of sex and violence in the association between substance use and depression.

**Fig. 3:**
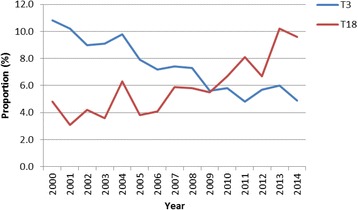
The dynamics of the hottest topic and the coldest topic from 2000 to 2014

The results of trend analysis on the proportion of the topics by year indicated that 4 topics, T9, T12, T17 and T18, showed a statistically significant increasing linear trend, and 4 topics, T3, T4, T6 and T15, showed a statistically significant decreasing linear trend. The dynamics of the hottest topic and the coldest topic, assessed by the coefficient of the trend test, are shown in Fig. 3. The hottest topic was T18, corresponding to brain research. The coldest topic was T3, corresponding to psychiatric disorders.

**Fig. 4:**
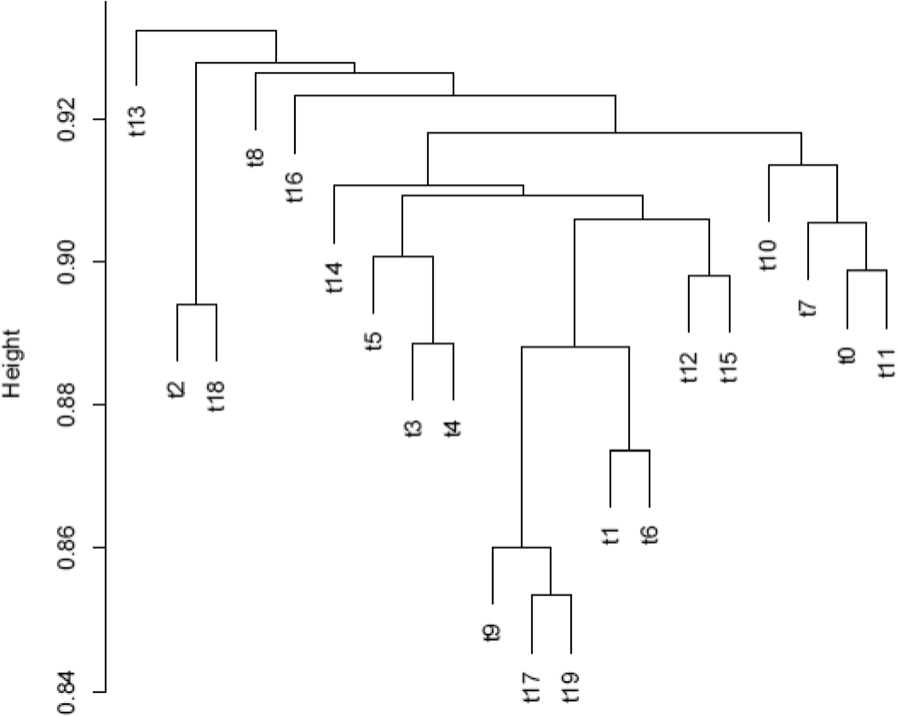
The cluster dendrogram of 20 topics based on hierarchical cluster analysis

The cluster dendrogram of 20 topics is shown in Fig. 4. The topics referring to related issues were clustered together because they contain many similar words. For example: T17 and T19 refer to risk factors which are associated with adolescent substance use or depression; T0, T7, T10, and T11 refer to substance use related issues; T12 and T15 refer to health care programs; and T3, T4, T5, and T14 refer to depression.

## Discussion

Literature review is fundamental and critical for understanding the current state of a theme, and it provides some direction for further study. In review articles that survey and summarize previously published material, authors often focus on a single specific issue with documentation appropriate for human reading and extracting. When the number of documents is large, text mining is a feasible way for information retrieval.

A way to evaluate the trustworthiness of the text mining algorithms is to check if the known facts could be found. The results indicated that some topics did recapture the search keywords regardless of the number of topics assumed in LDA. In addition to finding the known topics, LDA discovered other relevant topics, such as risk factors of adolescent substance use, the association link between adolescent substance use and adolescent depression, and intervention program.

Based on the results of LDA with different number(s) of topics, assuming too few topics misses meaningful issues and fails to segregate some distinct issues, and assuming too many topics may result in different topics referring to the same issue. A recent study empirically determined the appropriate number of topics by which model generated more relevant topics to the interested characteristics rather than by perplexity-based method [33]. Human judgment on the number of topics provides important insight.

One of the most popular topics among overlapped documents, which can be searched by adolescent substance use and adolescent depression, is T7 referring to sex and violence. We choose the subset of collections corresponding to documents assignment with T7 for further review. Sexual experience and violence are associated with adolescent substance use as well as with adolescent depression [34–36]. The link between sexual orientation and alcohol use may be mediated by depressive symptoms among sexual minority adolescents [37]. Depression mediated the association between bullying victimization and substance use among females [38]. Sexual assault during childhood increases the risk of depression and substance use, especially among Hispanic women [39]. These studies indicated that sex and violence play important roles in adolescent substance use and adolescent depression, and topic modeling performed in this study uncovered this meaningful issue.

Another popular topic among overlapped documents was T17, referring to the development from childhood to adulthood. We reviewed the articles corresponding to topic T17 and found some interesting results. The current longitudinal studies explored the reciprocal relation between substance use and depression [10–13], and discussed the influence of modifiers, such as personality, which moderated this association [40]. Adolescent alcohol use increased risk of later depression [41]. Adolescent depression increased the risk of later substance use [42, 43] and dependence on alcohol and nicotine [44, 45]. This literature provides implications for prevention programs. The substance use prevention programs should target adolescents with early psychiatric symptoms.

In literature review, one important issue is to analyze the dynamics of study subjects over time. Dynamic topic models [46] respect the ordering of the documents chronologically, assume a topic is a sequence of distribution over words which may change over time, and track how topic content, the most likely words, evolves over time. Assuming a single distribution over words for a given topic, this study aimed to explore the dynamics of proportion of a topic over time. We performed a post hoc analysis based on highest probable topic assignment of documents published during different years, and found that brain research is growing with the rapid development of technical advances in the neurosciences. Understanding the brain mechanisms, such as dopamine receptors, with neuroscience research could offer insights for medical treatment and behavioral prevention and assist in policy making [47].

LDA makes the “bag of words” assumption that words are generated independently from each other; hence, it does not consider word order. Some studies [48, 49] relaxed the bag of words assumption to extract the information of phrases in language generation. The main goal of this study was to uncover the course semantic structure of the texts, so we applied LDA to perform text mining. LDA captured correlations among words, but it cannot capture the correlation between different topics. Some studies [50, 51] have extended LDA and relaxed the assumption of independence of topics to explore the relationships between topics. This study first segregated a corpus into distinct themes using LDA. Subsequently, we performed post hoc analyses, hierarchical clustering on topics extracted by LDA, to explore the relationship between topics and to cluster similar topics. More studies are needed to explore whether the LDA-extended model provides additional insight on real data.

The volume of published articles are increasing at a considerable rate, hence literature mining and text mining methods are becoming essential to researchers to identify relevant studies, information extraction, hypothesis generation, et al. It was noticed that most of the text mining researches focus on titles, abstracts, or MeSH terms, yet much scientific information was harbored in full text, with limited access due to copyright restrictions. In addition, the bag of words ignores the order of the information in documents, though this approach has been popular recently since it is easily understood and applied. Considering a concept as a basic unit, conceptual mapping, instead of words, may provide additional insight [20]. Data mining approaches have great potential for knowledge discovery by integrating various information from text mining of literatures with other biological data [22].

## Conclusions

This study applied the LDA for text-mining of a vast amount of literature on adolescent substance use and adolescent depression. The results showed the ability of topic modeling to segregate a large collection of articles into distinct themes, and demostrated its usefulness as a tool to understand the literature by discovering relevant topics in addition to recapturing known facts. We performed trend analysis to identify the hot and cold topics, and hierarchical clustering analysis to cluster similar topics. We demonstrated the usefulness of topic modeling as a research tool to structure document collections and select a subset of documents on a particular topic to study in depth.

## Additional files

Additional file 1: Figure S1. The 10 most probable words in the topics of LDA with 5 topics. (PDF 126 kb) Additional file 2: Figure S2. The 10 most probable words in the topics of LDA with 20 topics. (PDF 367 kb) Additional file 3: Figure S3. The 10 most probable words in the topics of LDA with 50 topics. (PDF 263 kb)
