# Supplementary material for: Text mining for identifying topics in the literatures about adolescent substance use and depression
Source: BMC Public Health. 2016 Mar 19;16:279. doi: 10.1186/s12889-016-2932-1 (PMC4799597; doi:10.1186/s12889-016-2932-1)
Supplement: Additional file 1: Figure S1. — The 10 most probable words in the topics of LDA with 5 topics. (PDF 126 kb) [file 12889_2016_2932_MOESM1_ESM.pdf]

topic p The most 10 probable words

0	0.07	alcohol	ethanol	exposure	brain	rats	nicotine	adult	age	levels	response
1	0.10	treatment	health	intervention	care	substance	patients	children	depression	based	interventions
2	0.15	depression	symptoms	depressive	disorder	anxiety	disorders	children	years	age	psychiatric
3	0.11	alcohol	substance	sexual	behaviors	behavior	family	drinking	age	youth	problems
4	0.14	smoking	tobacco	alcohol	students	school	health	ci	years	prevalence	age

p: cumulative probability of the most 10 probable words
