# Supplementary material for: Text mining for identifying topics in the literatures about adolescent substance use and depression
Source: BMC Public Health. 2016 Mar 19;16:279. doi: 10.1186/s12889-016-2932-1 (PMC4799597; doi:10.1186/s12889-016-2932-1)
Supplement: Additional file 2: Figure S2. — The 10 most probable words in the topics of LDA with 20 topics. (PDF 367 kb) [file 12889_2016_2932_MOESM2_ESM.pdf]

| topic | p | The most 10 probable words | | | | | | | | | |
|---|---|---|---|---|---|---|---|---|---|---|---|
| 0 | 0.35 | substance | drug | abuse | alcohol | marijuana | cannabis | users | drugs | dependence | substances |
| 1 | 0.17 | school | students | health | ci | prevalence | girls | boys | age | years | high |
| 2 | 0.08 | asthma | children | disease | levels | blood | years | patients | age | serum | exposure |
| 3 | 0.22 | disorders | disorder | psychiatric | adhd | sleep | depression | anxiety | children | symptoms | clinical |
| 4 | 0.17 | treatment | depression | children | trials | patients | therapy | medication | placebo | clinical | controlled |
| 5 | 0.17 | patients | pain | depression | children | anxiety | life | group | psychological | chronic | years |
| 6 | 0.08 | research | health | review | prevention | interventions | social | young | based | development | people |
| 7 | 0.23 | sexual | behaviors | violence | sex | hiv | behavior | health | abuse | victimization | youth |
| 8 | 0.29 | children | child | problems | parents | family | mothers | maternal | parental | parent | offspring |
| 9 | 0.32 | depression | symptoms | depressive | anxiety | stress | girls | levels | depressed | coping | social |
| 10 | 0.42 | alcohol | drinking | consumption | related | binge | heavy | age | drinkers | drink | frequency |
| 11 | 0.32 | smoking | tobacco | smokers | cigarette | exposure | smoke | cigarettes | current | cessation | nicotine |
| 12 | 0.18 | intervention | treatment | program | group | based | follow | participants | control | interventions | months |
| 13 | 0.19 | weight | american | physical | eating | body | ethnic | health | activity | obesity | girls |
| 14 | 0.13 | scale | factor | scores | validity | depression | analysis | items | test | reliability | version |
| 15 | 0.22 | health | care | mental | services | treatment | patients | screening | primary | medical | problems |
| 16 | 0.25 | suicide | suicidal | ideation | ptsd | attempts | depression | trauma | injury | behavior | harm |
| 17 | 0.15 | age | early | adulthood | years | longitudinal | young | time | onset | genetic | adult |
| 18 | 0.09 | ethanol | brain | rats | exposure | adult | nicotine | response | memory | stress | mice |
| 19 | 0.16 | peer | social | family | substance | school | parental | behavior | behaviors | youth | perceived |

p: cumulative probability of the most 10 probable words
