# Supplementary material for: Text mining for identifying topics in the literatures about adolescent substance use and depression
Source: BMC Public Health. 2016 Mar 19;16:279. doi: 10.1186/s12889-016-2932-1 (PMC4799597; doi:10.1186/s12889-016-2932-1)
Supplement: Additional file 3: Figure S3. — The 10 most probable words in the topics of LDA with 50 topics. (PDF 263 kb) [file 12889_2016_2932_MOESM3_ESM.pdf]

topic	p	The most 10 probable words									
0	0.25	family	substance	parental	behaviors	behavior	protective	parenting	youth	parents	monitoring
1	0.53	smoking	tobacco	smokers	cigarette	cessation	smoke	cigarettes	nicotine	smoked	current
2	0.15	trials	review	interventions	search	included	analysis	quality	articles	literature	criteria
3	0.22	gene	genetic	allele	genotype	httlpr	interaction	polymorphism	rs	genes	environment
4	0.18	treatment	placebo	scale	weeks	depression	children	patients	week	clinical	response
5	0.29	internet	gambling	addiction	caffeine	music	video	online	energy	problem	media
6	0.41	depression	depressed	mdd	depressive	major	mood	disorder	group	clinical	onset
7	0.48	symptoms	depression	depressive	anxiety	levels	girls	relationship	high	boys	higher
8	0.23	intervention	program	group	control	based	prevention	trial	participants	follow	school
9	0.26	stress	depression	coping	life	events	cognitive	psychological	emotional	distress	attachment
10	0.51	substance	drug	marijuana	alcohol	cannabis	drugs	users	abuse	substances	illicit
11	0.22	care	screening	health	primary	patients	medical	depression	counseling	adherence	pediatric
12	0.21	ethanol	rats	exposure	adult	alcohol	mice	kg	days	animals	etoh
13	0.41	children	problems	parents	child	family	parent	parental	mothers	maternal	families
14	0.11	brain	expression	long	exposure	receptor	term	cell	cortex	cells	hippocampal
15	0.21	sleep	memory	cognitive	performance	insomnia	task	functioning	attention	deficits	verbal
16	0.23	treatment	depression	children	medication	patients	antidepressants	therapy	antidepressant	medications	efficacy
17	0.17	patients	years	age	injury	hospital	cases	ed	injuries	group	emergency
18	0.31	disorders	disorder	psychiatric	anxiety	children	diagnostic	mental	dsm	clinical	diagnosis
19	0.27	violence	abuse	victimization	sexual	physical	aggression	substance	maltreatment	dating	violent
20	0.25	eating	diabetes	body	depression	acne	weight	control	patients	qol	nervosa
21	0.10	research	prevention	based	interventions	youth	development	review	article	literature	important
22	0.24	treatment	substance	therapy	cbt	follow	outcomes	abuse	change	family	behavioral
23	0.14	genetic	behavior	environmental	personality	latent	class	trajectories	growth	high	differences
24	0.27	age	years	early	young	onset	adulthood	time	longitudinal	adult	follow
25	0.51	suicidal	suicide	ideation	attempts	depression	behavior	harm	suicidality	attempt	thoughts
26	0.12	patients	disease	blood	heart	cardiovascular	pressure	children	hypertension	adults	syndrome
27	0.22	ci	odds	regression	logistic	survey	ratio	prevalence	analyses	adjusted	past
28	0.42	adhd	disorder	sud	bipolar	hyperactivity	attention	deficit	cd	conduct	disorders
29	0.27	american	youth	african	ethnic	hispanic	white	differences	substance	groups	black
30	0.33	ptsd	stress	trauma	traumatic	symptoms	exposure	posttraumatic	children	depression	disorder
31	0.26	girls	boys	behaviour	age	health	years	gender	school	differences	prevalence
32	0.12	intake	bone	levels	serum	diet	dietary	concentrations	age	women	breast
33	0.32	physical	weight	activity	body	obesity	health	overweight	bmi	behaviors	exercise
34	0.26	exposure	asthma	children	smoke	tobacco	ets	cancer	cotinine	environmental	exposed
35	0.23	tobacco	exposure	smoking	media	advertising	youth	cigarettes	products	control	cigarette
36	0.41	health	mental	problems	services	care	youth	service	outcomes	physical	young
37	0.58	alcohol	drinking	consumption	related	problems	age	drink	expectancies	frequency	motives
38	0.21	social	peer	perceived	school	friends	support	influence	variables	model	peers
39	0.21	pain	depression	anxiety	children	patients	group	scores	chronic	life	quality
40	0.40	dependence	alcohol	abuse	substance	disorders	aud	dsm	criteria	iv	drug
41	0.24	school	students	high	prevalence	schools	survey	health	questionnaire	males	cross
42	0.13	brain	functional	matter	imaging	regions	magnetic	activation	resonance	left	cortex
43	0.12	young	people	parents	social	attitudes	participants	group	knowledge	groups	adults
44	0.46	drinking	alcohol	binge	heavy	driving	drinkers	college	related	behaviors	high
45	0.16	scale	factor	validity	scores	items	analysis	reliability	measure	version	item
46	0.33	sexual	hiv	sex	behaviors	behavior	intercourse	partners	sexually	risky	condom
47	0.34	pregnancy	exposure	maternal	prenatal	mothers	women	birth	pregnant	offspring	exposed
48	0.18	nicotine	stress	cortisol	levels	rats	day	hpa	response	axis	mg
49	0.12	development	behavior	early	behavioral	brain	developmental	reward	cognitive	affect	pubertal

p: cumulative probability of the most 10 probable words
